# De Quervain’s Disease: A Discourse on Etiology, Diagnosis, and Treatment

**DOI:** 10.7759/cureus.38079

**Published:** 2023-04-24

**Authors:** Adegbenro O Fakoya, Martin Tarzian, Enrique L Sabater, Daiana M Burgos, Gabriela I Maldonado Marty

**Affiliations:** 1 Cellular Biology and Anatomy, Louisiana State University Health Sciences Center, Shreveport, USA; 2 Psychiatry, University of Medicine and Health Sciences, Basseterre, KNA; 3 Anatomy, University of Medicine and Health Sciences, Basseterre, KNA

**Keywords:** eichnecoff, finklestein, wrist hyperflexion and abduction of the thumb test (what), first dorsal compartment, extensor pollicis brevis, abductor pollicis longus, stenosing tenosynovitis, de quervain’s disease

## Abstract

Since Fritz De Quervain first postulated stenosing tenosynovitis within the radial dorsum of the wrist, much research has been conducted to provide further insights. De Quervain’s Disease (DQD) is a condition that affects the tendons that control the movement of the thumb, specifically the abductor pollicis longus and extensor pollicis brevis. Numerous studies have shown that structural divergence from normal anatomy is partly related to contingency for developing DQD. Even though this condition was discovered many years ago, its exact etiology remains a subject of debate. Two schools of thought exist, one that contends an inflammatory-mediated pathway and the other degenerative changes. Substantial evidence exists for both theories, thus necessitating further studies into the etiology of DQD. Classically, Finkelstein’s and Eichhoff’s tests have been used as the physical examinations of choice for clinically diagnosing this condition. However, these tests have been shown to have low specificity, hence, the emergence of the wrist hyperflexion and abduction of the thumb test. Evidence also suggests that ultrasonography may become a critical diagnostic tool, especially to identify anatomical variations before invasive treatment, reducing the risk of further complications. The management of DQD is typically conservative, with escalation to steroid injections before surgery is indicated. Future research into this disease should focus on establishing a clearer picture of how anatomical variations and other pathological and occupational factors may interplay to bring about this condition. While current research has suggested possible novel approaches for diagnosing and treating DQD, more studies are required to gain greater insights into the effectiveness of these interventions.

## Introduction and background

De Quervain’s tenosynovitis, also known as De Quervain’s disease (DQD), is a painful condition that affects the tendons on the lateral side of the wrist. It is caused by inflammation of the tendons that control the movement of the thumb, specifically the abductor pollicis longus (APL) and extensor pollicis brevis (EPB) [1]. These tendons run through a narrow tunnel known as the wrist’s first extensor compartment. It is a fibro-osseous sheath that becomes constricted and inflamed in De Quervain’s tenosynovitis [1]. Thus, DQD can be defined as a stenosing tenosynovitis of the hand’s first radio-dorsal compartment containing the APL and EPB tendons [1,2]. It typically affects adults, most commonly women between 30 and 50 years of age [3], especially those who use repetitive hand or wrist motions in their daily activities [4,5]. However, it can also occur in men and women of any age group who engage in activities that strain the tendons in the wrist and hand, such as playing sports or using hand tools for extended periods [4-6].

Fritz De Quervain first reported this phenomenon in 1895 [7]. He theorized that this condition resulted from the repetitive strain among laborers who bore jobs onerous on the wrist (i.e., assembly workers) [7]. The condition typically causes pain and tenderness on the lateral side of the wrist and may radiate up the forearm. Patients may also experience swelling and difficulty moving the thumb or grasping objects [7]. Activities that involve repetitive hand and wrist movements, such as grasping, twisting, or pinching, can aggravate the condition [7].

The preliminary literature suggests that DQD results from myxoid degeneration rather than an underlying inflammatory process [5,8]. On the contrary, present-day investigation emphasizes the inflammatory markers that may cause a predisposition to this condition [3,8,9]. Other risk factors have recently been noticed, including somatotropin exposure and genetic propensity [10]. Anatomical variations of the first dorsal extensor compartment have been identified in numerous trials, and these variations have been shown to influence treatment outcomes [11]. As a result, the anatomical variation has been presumed to show inconsistent success rates via different therapeutic regimens [12,13].

The treatment methods, including physical therapy, corticosteroid injections, and therapeutic ultrasound, may need to be tailored to each patient’s unique wrist anatomy [12,13]. Paralleling the effectiveness of these therapies in extensive patient populations has provided insights into both the pathogenesis and ever-evolving approach to treating DQD [10-14]. We evaluate the present literature to provide a contemporary perspective on De Quervain’s tenosynovitis and elucidate the ongoing debate about its etiology, anatomy, diagnosis, and treatment.

## Review

Anatomical variations of the first dorsal compartment and clinical significance

The muscles in the human thumb are unique in evolutionary terms, and as a result, the anatomy of the first dorsal compartment can vary significantly between individuals [11]. The impact of anatomical variation on the likelihood of developing DQD is a fundamental point of interest and may explain why some individuals are more susceptible to developing this condition.

Here, we summarize the relationship between anatomical variation and the propensity for developing DQD. De Quervain’s tenosynovitis is more common in women than men, especially in those who are pregnant or have recently given birth [3]. It is also more prevalent in people who engage in repetitive hand and wrist movements, such as typing, knitting, gardening, or playing sports such as golf or tennis [4-6]. People with certain medical conditions, such as rheumatoid arthritis or diabetes, may also be at a higher risk of developing this condition. However, it can affect anyone who engages in activities that strain the tendons on the thumb side of the wrist [4,5].

Anatomical Variation and Propensity for De Quervain’s Disease

**Figure 1:**
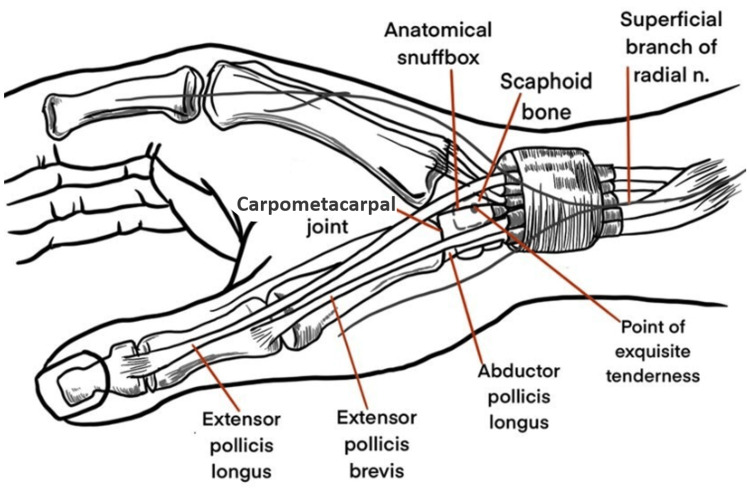
Schematic of the dorsolateral aspect of the wrist. This figure shows the anatomical location of the extensor pollicis brevis and abductor pollicis longus.

The standard arrangement of the first dorsal extensor compartment of the wrist comprises one APL tendon inserted into the base of the first metacarpal bone and one EPB tendon inserted into the proximal phalanx, as shown in Figure 1. An investigation conducted on patients with DQD commonly reveals anatomical variation which differs from the standard anatomical model described above [12-14]. These variations tend to increase the likelihood of developing this condition and the risk of pain recurrence and complications after invasive treatment procedures.

One prevalent anatomical variant seen in many DQD patients is the presence of complete or incomplete septa within the first dorsal compartment. This septum is a fibrous tunnel that extends through the compartment and divides the APL tendon. In contrast, the EPB tendon slips into different subcompartments at the level of the radial styloid [11,15]. The sub-compartmentalization of these tendons not only compresses the local structures of the wrist dorsum, thus causing inflammation but also commonly results in inadequate decompression of tendon inflammation during surgical treatment, leading to the eventual relapse of symptoms [16-18].

Various studies have reported different findings regarding the number of tendon slips in the APL and EPB muscles across different patients and cadavers [17-19]. In most studies that involved surgical patients, the APL tendon typically ranges from one to six tendon slips. In studies involving cadavers, only two slips of this tendon are usually observed. Recent research, however, has shown that a single APL tendon slip is more frequently observed in patients with DQD during surgery [19]. In contrast, the EPB tendon is commonly described as a single tendon slip in most cases. The absence of an EPB tendon has also been illustrated in some patients, and very rarely have there been cases identified with multiple EPB slips. The most common anatomical combination of tendon slips between normal cadavers and DQD patients includes one EPB tendon compartment (considering potential accessory slips) and two APL tendon slips [17,19]. However, among the compartments analyzed, only DQD patients presented more consistently with one APL tendon and one EPB tendon slip separated into subcompartments by a septum. Different insertion sites result in different gliding resistances or friction coefficients, which potentially predispose certain individuals to stenosing tenosynovitis of the first dorsal compartment [20,21].

Etiology of De Quervain’s disease: an overture

The exact etiology of DQD is a subject of investigation; many prevailing theories have arisen since De Quervain first postulated its origin in his preliminary paper [7]. He speculated that it is an occupational disease augmented through industrial expansion and the increased demand for repetitive wrist motions to complete assembly production [7]. A cross-sectional study conducted in 2011 supports this theory. When 3,710 factory workers from France were examined between 2002 and 2005, logistic regression modeling revealed that work involving pronation and supination of the wrist (i.e., screwdriving) was associated with DQD [22]. Although the original postulation by De Quervain remains popular, its merit has been called into question by several classic publications. A quintessential paper by Thomas and his colleagues studied peritendinitis in 544 Luton Car Plant workers between the 1940s and 1950s. It disclosed that there were only two recorded cases of DQD in this group, which was not more prevalent than in the common population [6].

Several explanations for the underlying cause of DQD have been proposed since its discovery. Furthermore, if labor-related microtrauma were the chief culprit behind this disease, it would be more prevalent in men who predominated the assembly lines. Yet, DQD is three times more common in women, particularly women of childbearing age [4]. Moreover, if DQD is indeed labor-related, it should show a proclivity for the wrist of the dominant hand, yet this has been shown to be false in several studies [4,5]. It would seem then, at best, work-related torsion is merely a catalyst for eliciting these symptoms. It may well be that rather than inflicting DQD, the industrial expansion elicited flares of existing cases that were previously inconspicuous. We will review and discuss the prominent etiological theories in this section.

Inflammation

Inflammatory mediators are molecules released during the inflammatory response and play a crucial role in the pathogenesis of DQD by promoting the recruitment of immune cells to the site of inflammation, increasing blood vessel permeability, and inducing pain [5,8]. These molecules also contribute to tissue damage by promoting the production of reactive oxygen species and promoting the activation of matrix metalloproteinases, which are enzymes that degrade the extracellular matrix of tissues [8-10]. Classically, researchers have accepted the pathogenesis of DQD to be angiogenic and fibrotic rather than inflammatory. Be that as it may, anti-inflammatory medication remains the universally accepted conservative treatment [17,23]. Thus, there are grounds for suspecting an underlying inflammatory process mediating the downstream cascade that leads to DQD.

Kuo and colleagues were the first to explore this avenue in 2015. Not only did they find inflammatory mediators (neutrophil elastase, macrophages, and COX-2) in specimens with DQD, but they also found a correlation between the severity of the symptoms and the expression of these mediators [8]. In addition, Kuo published a study showing increased expression of the inflammatory mediators interleukin (IL)-20 and tumor necrosis factor-alpha (TNF-α) in patients with DQD [9].

The inflammatory pathways may also explain the propensity of DQD for the female sex. Shen et al. revealed that estrogen-B receptors (ER-Bs) (inducers of COX-2 expression) are expressed with greater magnitude in DQD patients, and greater expression is correlated with more severe symptomatology [3]. Thus, an estrogen surge may explain the predilection of DQD for women of childbearing age and menopause. With further research, therapeutic plans that target ER-B may alleviate these symptoms, particularly in women. Furthermore, a study by Shen et al. demonstrated the expression of inflammatory factors IL-1B, IL-6, vascular endothelial growth factor, and Von Willebrand factor, which were also positively correlated with symptom severity [3]. Shen et al. hypothesized that macrophagic invasion into the synovial membrane might induce the generation of inflammatory factors, which result in chronic inflammation and angiogenesis [3].

Other findings in support of the inflammatory theory include the demonstrable presence of IL-1β and IL-6, which are elevated in the tenosynovial tissue of patients with DQD [3,9]. Additionally, prostaglandins, lipid mediators that play a role in inflammation and pain, have been found in high levels in the tenosynovial tissue of patients with De Quervain’s tenosynovitis [3]. Thus, it is undeniable that inflammation plays a critical role in the pathogenesis of DQD. As future research sheds light on these specific pathways, treatment must adapt to target them accordingly.

Chronic Myxoid Degeneration

The presence of inflammatory activity within DQD specimens has been demonstrated in multiple studies. With so much evidence, it is enticing to assume that inflammation within the synovium and tendon sheath gives rise to DQD and not further inquire about its etiology [5,6]. However, some studies suggest inflammation may be a confounding variable that cloaks the true pathological nature of this condition [5,6]. It has been argued by some classic publications that the inflammatory components of DQD are merely superimposed on myxoid degeneration occurring within the tendon sheath and synovium [5,6].

Clarke et al. examined the tendon sheaths of 23 patients with DQD and observed notable increases in vascularity and mucopolysaccharides compared to controls [6]. Furthermore, only four of the 23 specimens in this study contained lymphocytes within the tendon sheaths, and no lymphocytes were found within the synovium of any test subjects [23]. Clarke et al. argued that although inflammation may be present in patients with DQD, it is not an etiological factor but rather an overlying process that masks myxoid degeneration [7]. Read and colleagues observed intramural deposits of mucopolysaccharides below the synovium of six women who developed DQD during pregnancy or within 12 months of childbirth [6]. Interestingly, not one specimen in their study showed any signs of inflammation [6].

Other Etiological Factors

DQD has been investigated from multifarious paradigms, providing insights into the factors contributing to its pathogenesis. A genome-wide investigation by Kim et al. in 2017 confirmed that a reference SNP cluster on chromosome 8 (rs35360670) is linked with DQD [24]. To our knowledge, this was the first study to demonstrate an association between allelic variation and DQD. Apart from genetic factors, a case study by Yurdakul et al. in 2017 reported a potential link between the administration of somatotropin treatment and the development of the condition. The study involved a 14-year-old girl. The pediatric patient presented a persistent DQD after being treated with somatotropin hormone therapy for growth hormone (GH) deficiency [25]. GH and insulin-like growth factor 1 levels are associated with musculotendinous collagen expression [25]. Increased collagen synthesis causes thickening of the flexor tendons and synovial edema, which may lead to tenosynovitis of the frequently used tendons of the hand [5]. A paper published by Lipscomb in 1951 argued that angulation on the radial side of the wrist is farther in female anatomy and may, therefore, partly explain why women are affected more than men [26].

Pathology: histopathology - what do the slides say?

In Clarke’s classic study, the tendon sheaths of DQD patients were shown to be five times thicker than in controls, with considerable dense mucinous deposition within the synovium. Exaggerated vascularity within the central portion of the fibrous sheath was also demonstrated. Of note, only four of the 23 patients in this study presented with lymphocytic infiltration in the tendon sheath. Interestingly, no histological support for an active inflammatory process underlying DQD was demonstrated in this study [22]. Hooper and colleagues examined the histological appearance of fibrous tendon sheaths in postpartum patients with DQD [5]. Like in Clarke’s study, histological examination revealed striking amounts of mucinous and myxoid degeneration within the synovium [5]. The tenosynovial regions of the specimens showed a marked accumulation of mucopolysaccharides.

Similar to the results found in Clarke’s study, the central portion of the fibrous sheath also demonstrated mild angiogenesis [5]. Hooper’s results also poorly showed evidence for an inflammatory-mediated pathway. In the same way as Clarke and Hooper, Kuo and colleagues found intense thickening of the fibrous tendon sheath with varying degradation of collagen structure and angiogenesis [8]. Unlike Clarke and Hooper, Kuo performed immunochemical staining, which revealed increased expression of inflammatory markers, including neutrophil elastase, macrophages, MAC387, and COX-2-positive cells [8]. Statistical analysis revealed that the expression of these inflammatory markers was positively correlated with the severity of symptoms (except MAC387, which was maximally expressed in moderate cases of DQD) [8].

Diagnostics

The Finkelstein and Eichhoff tests are commonly used in clinical practice to diagnose DQD [27]. The Finkelstein test involves the examiner holding the patient’s thumb firmly with one hand while applying firm traction longitudinally and in the direction of slight ulnar deviation to the wrist with the other hand. In contrast, the Eichhoff test requires the patient to oppose the thumb into the palm and clench the fingers while the examiner passively applies ulnar deviation to the wrist [28]. Despite being effective, there are arguments against these methods due to false-positive results and examination discomfort. Such findings are ascribed to the fact that they are passive tests that have the disadvantage of stressing different structures that are not directly involved in the pathology of DQD. A study from 2018 demonstrated that, although both tests have these limitations, the Finkelstein test has a higher specificity and fewer false positives when compared to the Eichhoff test [27,28]. In that study, the recorded specificity for the Finkelstein and Eichhoff tests was 100% and 89%, respectively [28]. Due to the controversy generated by these tests, a new active diagnostic strategy called the wrist hyperflexion and abduction of the thumb test (WHAT) has emerged. During WHAT, the patient is asked to actively hyperflex the wrist and abduct their thumb as the examiner’s index finger provides counter pressure, which will elicit pain if DQD is present [27,28]. This test identifies DQD exacerbation while minimizing shear between APL/EPB and the bony floor of the first extensor compartment [27,28]. In addition to these benefits, this test has been proven to be more accurate in diagnosing DQD, as stated by another study in which both specificity and sensitivity values were significantly higher for the WHAT test when compared to the Eichhoff test. In that discussion, the specificity was 14% for the Eichhoff test and 29% for the WHAT test. Furthermore, the sensitivity for both tests was recorded as 89% and 99%, respectively [28].

Most studies suggest that patients with septation in the first dorsal extensor compartment of the wrist tend to develop De Quervain’s and have post-treatment complications [29]. An excellent diagnostic tool for identifying this septation before treatment is ultrasonography. Some studies have focused on proving the efficacy of ultrasonography in detecting both APL/EPB tendons, their sizes, and the presence or absence of a septum between them. In Nagaoka’s clinical study, preoperative ultrasonography was done on 32 wrists of patients with DQD, and it successfully identified septation in 25 of them before their surgeries [30]. This method could be beneficial in diagnosing De Quervain’s and identifying the possible anatomy of the patient’s wrist before executing the treatment plan to reduce the risk of postoperative complications significantly. Some limitations to this tool do exist. First, the physician must know that a septum will usually present as a hypoechoic area in ultrasonography. Other lesions, such as intratendinous degeneration, synovial proliferation, or fluid, could also be perceived as a hypoechoic area and should be differentiated [29,30]. Overall, ultrasonography could be the key to identifying anatomic variations, which may aid in decreasing the incidence of post-treatment complications and regression of symptoms [29].

Treatment

Current De Quervain’s Disease Treatment and Rehabilitation Methods

Once a physical examination concludes with a newly diagnosed DQD patient, the following steps for treatment are divided between a multitude of non-surgical approaches and the last-resort surgical approach if symptoms fail to subside. Current non-surgical methods commonly include hand physical therapy, thumb spica splints to immobilize the irritated tendons, anti-inflammatory non-steroidal anti-inflammatory drug (NSAID) prescriptions, and corticosteroid injections to reduce the inflammatory swelling and irritation of the APL/EPB tendons. Although these non-surgical approaches solve the concerns of immediate pain, there remains a considerable incidence of pain recurrence, which has borne the question of how efficient are these methods for curing DQD patients. It was initially understood that corticosteroid injections alone had almost a six times greater cure rate than splints alone [31,32]. Later studies exploring the efficacy of comparing individual versus combined non-surgical approaches further illustrate that multimodal treatment plans of hand therapy with corticosteroid injection minimally reduce visual analog scale pain scores more than using the steroid injection method exclusively [13,14]. To date, steroidal injections directly proximal to the radial styloid process remain the treatment of choice for newly diagnosed DQD patients [17]. Initial corticosteroid injections have proven a cure rate ranging from 62% to 100% with the failure-to-cure associated with a present APL/EPB septum or specific mechanical triggering of the first dorsal compartment [32,33]. A second injection is usually administered for patients with pain recurrence two weeks after the first injection. If pain remains two weeks later, then it is expected that a third injection would be futile to alleviate the symptoms, therefore, requiring a surgical approach [33,34].

​​Another non-surgical physical agent that can be utilized is therapeutic ultrasound. Therapeutic ultrasound is a rehabilitative modality used for different musculoskeletal injuries to enhance tissue extensibility, reduce pain, and improve healing in wounds, tendons, and bones [35]. It is based on high-frequency sound waves at varying parameters depending on the presenting condition and treatment goals [34-36]. A 3 MHz frequency is applied for superficial structures and is commonly used for DQD [35]. Once swelling and pain have been treated, therapeutic exercises can be incorporated. This approach is based on performing different range of motion (ROM) exercises that enhance the gliding of the APL and EPB tendons in the first dorsal compartment, starting with isometrics and then completing ROM against gravity [36-38]. Another mechanism for rehabilitation is therapeutic Kinesio taping (KT). It focuses on positioning Kinesio tapes to release interstitial pressure and reduce inflammation [37]. To achieve a diminished contraction, KT must be applied from the insertion of the muscle to its origin [37]. It is presumed to allow the decompression of subcutaneous nociceptors to reduce pain [37].

A surgical release of the first extensor compartment is required for extreme cases of DQD patients failing to resolve symptoms within six months of corticosteroid injections or other non-surgical treatments. When accessing the first dorsal compartment, it is pivotal to longitudinally incise the EPB subcompartments (the tendon most likely needing decompression) and the septum dividing APL/EPB tendons if present [18]. Failure to properly incise the septum will ultimately result in failed decompression and refractory DQD symptoms. Another complication during the surgical release is EPB tendon subluxation which can be prevented by avoiding complete excision of the EPB tendon sheath [34,35]. This solution to tendon subluxation does not commonly apply to the APL tendon as dorsal decompression avoids damaging the tendon sheath surface toward the EPB [36]. After the procedure, postsurgical intervention recommends a 1-2-week thumb spica splint followed by weeks of active range-of-motion exercises, scar/edema management, and strengthening exercises [13].

Consequences of Anatomical Variations on Steroid Injections and Surgery

Studies have shown that a consistent incidence of DQD patients remains for those having pain recurrence after the initial injection and thus require a second corticosteroid injection [17,38]. Interestingly, most DQD patients receiving a second corticosteroid injection do not require a third dosage to resolve their symptoms [38,39]. The necessity of the second dosage is commonly attributed to the anatomical variations between DQD patients. The injection site and subcompartments or septation are crucial factors that must be considered for both the efficacy of corticosteroid injections and the surgical release of the first dorsal compartment. Across multiple studies, the variation of the septum beginning at the radial styloid and dividing APL/EPB tendons at the first dorsal compartment has been commonly theorized to enclose the initial injection to only one tendon compartment, therefore, leaving the other tendon untreated and allowing for symptoms to resurface. Consequently, it is common practice for the second injection to target the potential EPB tendon subcompartment dorsally as it is most likely to be more irritated than the APL [38].

As mentioned previously, surgical release of the first dorsal compartment for refractory DQD patients requires severing the EPB subcompartment and the septum, if present, to ensure a successful procedure. Interestingly, reporting shows that approximately 62.2% of surgically treated DQD patients have a septum; among those, only approximately 58.5% are an incomplete septum [17]. The lack of adequately making a transverse incision at these two structures results in failed decompression of the first dorsal compartment and a recurrence of pain weeks later [39-41]. It has also been noted that DQD patients present with a greater subcompartment incidence, complicating the surgical release procedure intending to cure the patient and avoid postoperative complications or failed treatment.

Modern Conservative Treatment Approaches

Here, we summarize unique novel therapeutic methods for tenosynovitis that can assist and even accelerate healing along the treatment plan for DQD patients, such as ultrasound, phonophoresis, iontophoresis, and the Graston technique. High 3 MHz frequency ultrasound has been found to be contraindicated for patients with acute inflammation or surgical tendon repairs within the last six weeks [40]. Phonophoresis/sonophoresis uses ultrasound to direct topical anti-inflammatory medications deeper into tissues. In contrast, iontophoresis uses an electrical current/gradient to deliver anti-inflammatory medication to shallow regions of the hands/feet to reduce edema, inflammation, scar tissue, and pain [40,41]. These techniques are commonly used for patients with hyperhidrosis but have also been helpful for chronic overuse tendinopathies and stenosing tenosynovitis such as DQD. It is also unclear if these methods of administration properly reach the desired tissues before being diluted by microvasculature. The Graston technique aims to cause controlled microtrauma to the desired soft tissue to augment mobilization and regeneration by following the principles of Wolf’s Law, referring to tissue remodeling per the stress placed on it [31,40-43]. With these new treatment approaches and more development, the DQD treatment timeline presents a trend enhancing the rehabilitation of stenosing tenosynovitis and reducing the time necessary for a patient to recover from persistent pain and regain strength. The most significant caveat of these practices is the need for more available research regarding the efficacy of such treatment and comparable utility to current surgical/non-surgical treatment methods.

## Conclusions

We showcased DQD, a stenosing tenosynovitis, that has generated debates regarding etiopathogenesis, diagnosis, and treatment. DQD is typically managed conservatively using oral NSAIDs, including physical therapy, splinting, therapeutic ultrasound approaches, and microtrauma assisted-healing techniques before resorting to corticosteroid injections or even surgery when indicated. Our review has shown that ultrasonography should be recommended as it might serve as the link to establishing a clearer picture of how anatomical variation may interplay with the pathological factors resulting in this condition and reduce the risk of postoperative complications. Overall, further investigation is necessary to refine and further elaborate our current understanding of DQD.
